# Histopathologic differences in granulomas of *Mycobacterium bovis* bacille Calmette Guérin (BCG) vaccinated and non-vaccinated cattle with bovine tuberculosis

**DOI:** 10.3389/fmicb.2022.1048648

**Published:** 2022-11-08

**Authors:** C. Kanipe, P. M. Boggiatto, E. J. Putz, M. V. Palmer

**Affiliations:** ^1^Infectious Bacterial Diseases Research Unit, National Animal Disease Center, Agricultural Research Service (USDA), Ames, IA, United States; ^2^Immunobiology Graduate Program, Iowa State University, Ames, IA, United States

**Keywords:** granuloma, *Mycobacterium bovis*, BCG, bovine tuberculosis, tuberculosis vaccine

## Abstract

Mycobacterium bovis (*M. bovis*) is the zoonotic bacterium responsible for bovine tuberculosis. An attenuated form of *M. bovis*, Bacillus Calmette-Guerin (BCG), is a modified live vaccine known to provide variable protection in cattle and other species. Protection for this vaccine is defined as a reduction in disease severity rather than prevention of infection and is determined by evaluation of the characteristic lesion of tuberculosis: the granuloma. Despite its recognized ability to decrease disease severity, the mechanism by which BCG imparts protection remains poorly understood. Understanding the histopathologic differences between granulomas which form in BCG vaccinates compared to non-vaccinates may help identify how BCG imparts protection and lead to an improved vaccine. Utilizing special stains and image analysis software, we examined 88 lymph nodes obtained from BGC-vaccinated and non-vaccinated animals experimentally infected with *M. bovis*. We evaluated the number of granulomas, their size, severity (grade), density of multinucleated giant cells (MNGC), and the amounts of necrosis, mineralization, and fibrosis. In one set of banked samples BCG vaccinates had fewer granulomas overall and lower numbers of multinucleated giant cells. In the other set of samples, lesions of vaccinates were significantly smaller. In both experimental groups vaccinates had less necrosis than non-vaccinates. The relative numbers of highand low- grade lesions were similar between vaccinates and non-vaccinates of both groups as were the amounts of fibrosis and mineralization. Collectively, these findings demonstrate the variability of protection offered by BCG. It suggests that BCG vaccination may serve to reduce bacterial establishment, resulting in the formation of fewer granulomas and in granulomas that form, that it may have a protective effect by containing their size and reducing the relative amount of necrosis. The amount of fibrosis was higher in low-grade granulomas from vaccinates compared to non-vaccinates. Collectively, these findings suggest that BCG vaccination reduces bacterial establishment, resulting in the formation of fewer granulomas. In granulomas that form, BCG has a protective effect by containing their size, reducing the relative amount of necrosis, and increasing fibrosis in low-grade lesions. Vaccination did not affect the amount of mineralization or density of MNGC.

## Introduction

*Mycobacterium bovis* (*M. bovis*) is the most host-promiscuous member of the *Mycobacterium tuberculosis* complex (MTBC), capable of causing tuberculous disease in over 85 species of animals, including some endangered wildlife species (Kanipe and Palmer, 2020). It is the primary cause of bovine tuberculosis (bTB) worldwide. It is a scourge to farmers, livestock, and conservationists by exacting economic tolls, as well as causing disease in both animals and humans.

Bacillus Calmette-Guérin (BCG) is an attenuated strain of *Mycobacterium bovis* which has been used as a vaccine for protection against *Mycobacterium tuberculosis* (Mtb) in humans for over 100 years. It has long been accepted as providing variable protection against virulent *M. bovis* in livestock and wildlife (Buddle et al., 2018; Roy et al., 2019). Importantly however, protection is often defined as a decrease in disease severity rather than protection from infection. Additionally, currently utilized diagnostic techniques based on purified protein derivative (PPD), also known as tuberculin, fail to differentiate BCG-vaccinates from *M. bovis* infected animals, further complicating both its use, and role in disease eradication and control efforts. Reports of protection range widely from 0 to 100% depending on age, route of vaccination, vaccine dose and uncontrollable variables such as genetics and environment (Canto Alarcon et al., 2013; Buddle et al., 2018; Bayissa et al., 2021). In naturally infected cattle results vary, but BCG vaccinates have been shown to have lower bTB prevalence (based on isolation of *M. bovis*), lower numbers of cattle with gross lesions, and less severe disease than non-vaccinates (Nugent et al., 2018; Bayissa et al., 2021). Because of its cross reactivity with the primary herd diagnostic, the tuberculin skin test, and the success of eradication programs in decreasing overall prevalence of bTB, interest in BCG vaccination in most countries decreased during the mid to late 20th century. Unfortunately, complete eradication has been unsuccessful in most countries for various reasons including, lack of sensitivity and specificity in current diagnostic assays, increases in the size of concentrated feeding operations (CAFOs), importation of infected animals from other countries and the presence of wildlife reservoirs of *M. bovis* with persistent wildlife-to-cattle transmission (de la Rua-Domenech et al., 2006; Fitzgerald and Kaneene, 2013; Ciaravino et al., 2021). Thus, there is a renewed interest in vaccines. Even with the shortcomings of BCG, thus far, no experimental bTB vaccine has consistently outperformed BCG in vaccine efficacy studies. Until a vaccine which consistently improves protection against disease is found, it is prudent to continue research on BCG’s properties and how it imparts protection. Numerous studies have explored the differences in peripheral responses between vaccinates and non-vaccinates, but this provides an incomplete picture of pathogenesis, as it is distant from the site of active host-pathogen interaction in the tissue. In order to fully elucidate how BCG works, we therefore need intralesional examination (Jones et al., 2017; Steinbach et al., 2021; Khalid et al., 2022).

The characteristic lesion of tuberculosis is the granuloma. Granulomas are collections of macrophages with multiple modifiers such as the presence or absence of varying amounts of neutrophils, lymphocytes, multinucleated giant cells (MNGC), fibrosis, necrosis, and mineralization. These are dynamic structures, with cell trafficking resulting from host- and bacterially-originating signals. Until recently, it was believed that the tuberculous granuloma was a structure which benefited both host and pathogen at different points in infection, early on by promoting dissemination of the bacteria as a result of migrating macrophages, and later by “walling off” the offending intruder through fibrosis. Although this paradigm remains popular, new advances in research create challenges for its defense (Ramakrishnan, 2012). Understanding the differences between granulomas that form in BCG vaccinates compared to non-vaccinates may not only help identify how BCG imparts protection but may also more aptly help define how it benefits pathogen as well as host. While tuberculous granulomas have been documented in numerous organs, the principal sites in cattle are the thoracic lymph nodes (tracheobronchial and mediastinal) and within the lung parenchyma. Interestingly, cattle and humans share histologic similarities in their granuloma structure only surpassed by non-human primates. Due to the financial and ethical implications with using non-human primates, this elevates the importance of work performed in cattle, which could yield valuable insight into human disease.

Previous pioneering works by Johnson, *et. al* have demonstrated reduced granuloma numbers, size, necrosis, peripheral fibrosis, and MNGC numbers in lymph nodes of BCG vaccinated animals compared to non-vaccinated controls when experimentally infected with virulent *M. bovis* (Johnson et al., 2006). The reduction in granuloma number and size, as well as decreased tissue destruction suggest improved host control over the bacteria and decreased MNGC numbers suggests a reduction in antigen persistence(Pagan and Ramakrishnan, 2018; Trout and Holian, 2020; Palmer et al., 2022). Decreased peripheral fibrosis is likely associated with decreased bacterial burden compared to non-vaccinates as demonstrated in non-human primates (Gideon et al., 2022). The aim of this study is to build, confirm and expand upon Johnson’s work in several key areas. Using histopathologic examination combined with advanced imaging software, we examine a total of 88 pulmonary lymph nodes from 22 BCG-vaccinated and 22 non-vaccinated cattle, all of which were experimentally infected with virulent *M. bovis.* The result of this study is a comprehensive look of the pathology that forms in the pulmonary lymph nodes of BCG-vaccinates and non-vaccinates.

## Materials and methods

### Samples

Banked tissue samples from two previous studies were utilized. In both studies cattle were obtained from bTB-free herds from Iowa, United States. Briefly, in Experiment 1 (E1), 23 castrated Holstein steers of 4–5 months of age were divided into two groups: non-vaccinates (*n* = 11) and BCG vaccinates (*n* = 12). Animals were kept on pasture prior to the start of the study. As environmental bacteria are present in our area, and to prevent possible confounding responses to BCG vaccination, animals were confirmed non-reactive to *Mycobacterium avium* (*M. avium*) *via* interferon gamma release assay (IGRA) immediately before the start of the study. Animals in the vaccinated group received a subcutaneous injection 1 ml of 5 × 10^5^ CFU of BCG Danish. Three months following vaccination, both vaccinates and non-vaccinates received 5.5 × 10^2^ CFU of *M. bovis* strain 10-7428 *via* aerosolization as described elsewhere (Palmer et al., 2002). Animals were euthanized 13–17 weeks post-challenge. In Experiment 2 (E2), 21 newborn Holstein steers were divided into two groups: non-vaccinates (*n* = 11) nd BCG vaccinates (*n* = 10). Animals were bottle-raised on a pasteurized milk product and kept in clean pens to reduce exposure to environmental mycobacteria. Due to the cleanliness of the housing situation the risk of exposure to environmental mycobacteria was considered low and IGRA testing for *M. avium* was not performed. Animals in the vaccinated group received a subcutaneous injection 1 ml of 1 × 10^6^ CFU of BCG Danish at 2 weeks of age. Three months following vaccination, both vaccinates and nonvaccinates received 1 × 10^3^ CFU of *M. bovis* strain 95-1315 via aerosolization. Approximately 18 weeks post-challenge animals were euthanized. In both studies animals were humanely euthanized by intravenous administration of sodium pentobarbital. All animal procedures were approved prior to the experiments by the Institutional Animal Care and Use Committee (IACUC) at the National Animal Disease Center. At necropsy, both experimental groups demonstrated a significant reduction in gross lesions in the lungs of BCG-vaccinates compared to non-vaccinates and a significant decrease in CFU/gram of lymph node tissue between vaccinates and non-vaccinates (Waters et al., 2009). An approximately 2.5 × 2 × 0.4 cm randomly selected sample of both the tracheobronchial and mediastinal lymph nodes were collected from each animal resulting in a total of 88 lymph node samples, 44 from each group. Samples were fixed in 10% neutral buffered formalin, paraffin embedded following standard techniques, and sectioned 4–5 μm thick prior to staining with Harris hematoxylin and eosin (H&E).

### Stains and scanning into HALO

Paraffin-embedded sections were stained with H&E using a Leica 5,020 multistainer (Leica/Surgipath). Von Kossa Calcium and Masson’s Trichrome staining were performed following manufacturer’s instructions (Newcomer Supply) for calcium and collagen fiber analysis, respectively. Following staining, slides were digitally scanned using the Aperio AT2 whole slide imager, to generate bright-field whole- slide images at 20X (0.5 μm/pixel) and 40X (0.25 μm/pixel) magnification in a 24-bit color pyramid TIFF (.SVS file). The. SVS files were transferred into HALO software (HALO v3.1.1076.291, Indica labs) for annotation and analysis.

The mediastinal and tracheobronchial lymph node values for each metric were initially evaluated separately to ensure differences were not a result of anatomic location. No significant differences were noted, therefore values from the tracheobronchial and mediastinal lymph nodes were combined and averaged to yield a single value per animal.

### Evaluation of granuloma number, grade, total size, necrosis area, cellular area, and number of MNGCs

Digitally scanned images were evaluated at 10X magnification. Using HALO annotation tools, granulomas were outlined in different colors based on grade, and the total granuloma area calculated using HALO’s annotation software. A granuloma was defined as a focus consisting of increased accumulations of macrophages admixed with varying numbers of lymphocytes, neutrophils and MNGCs. If a tissue section lacked granulomas, it was excluded from the remaining histopathologic examinations. Granulomas were separated into two grades: low and high.

Low-grade granulomas were defined as singular, with approximately ≤10% necrosis. High-grade granulomas were defined as having greater than 10% of its total area as necrosis, being multicentric, or coalescent with other granulomas. Necrosis was defined as an area of tissue destruction characterized by degenerating, fragmented cells, and structural debris with or without mineralization. When present, fibrous encapsulation was included in the total granuloma area calculated. Care was made to closely follow the directional flow of the collagen fibers when numerous, closely associated but independent granulomas were present. When fibrous bands incompletely separated granulomas or had no identifiable pattern of separation, these were counted as a single, coalescing lesion and considered high grade.

**Figure 1:**
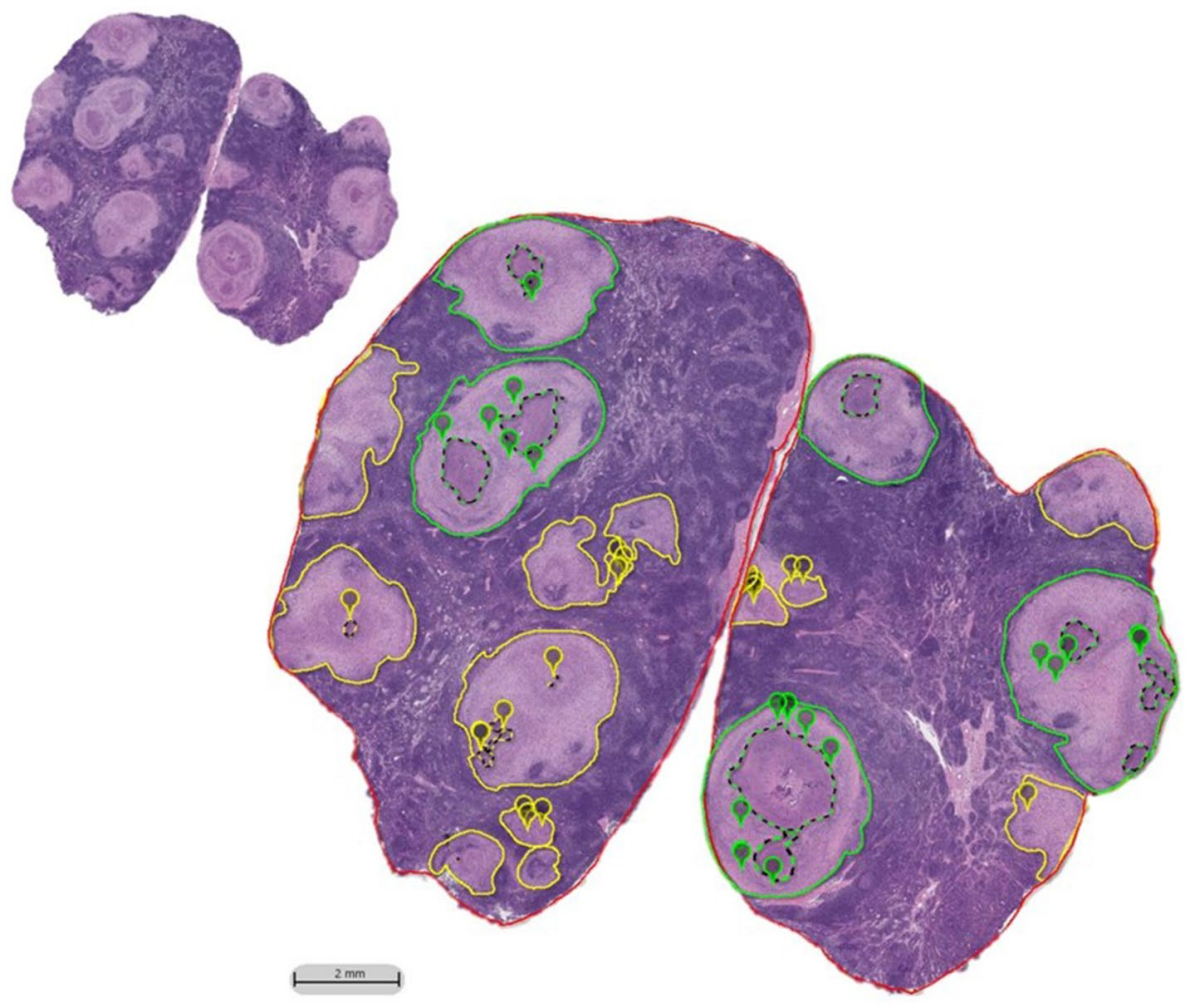
HALO imaging annotations performed on H&E tracehobronchial lymph node sections of BCG-vaccinated and non-vaccinated cattle challenged with aerosolized *M. bovis*. Granulomas were outlined in either green or yellow based on the amount of necrosis and/or if their boundaries were indistinguishable from the neighboring granuloma (coalescing). If there was ≤10% necrosis, it was classified as a low-grade granuloma and outlined in yellow. If there was >10% necrosis or if it was a collection of coalescing granulomas, it was outlined in green and categorized as high-grade. Necrosis, when present, was outlined by a dotted line of a matching color. MNGCs identified at 10X were marked with a pin of the same color as the grade designation. H&E section prior to annotation shown in inset.

High grade granulomas were outlined in green while low grade granulomas were outlined in yellow (Figure 1). The absolute number and the number of each type of granuloma were enumerated and totaled for each animal. For high- and low-grade granulomas, the average granuloma size was calculated by dividing the total granuloma area within an animal by the number of granulomas of that grade. Necrosis was outlined using a dotted line of the corresponding grade color, and its area summated to provide the total area of necrosis. The percent necrosis was calculated, and if a low-grade granuloma was found to contain ≥10% necrosis, it was redefined as a high-grade granuloma. Enumeration of MNGCs was performed by placement of digital pins which were summated (see Figure 1). Multinucleated giant cells were defined as macrophages containing three or more nuclei within a continuous cytoplasm, identifiable at 10X magnification, in accordance with other published studies(Trout and Holian, 2020; Losslein et al., 2021).The density of MNGCs was calculated as the number of MNGC present per 100μm^2^ of non-necrotic area.

Numerous previous studies utilized a four-stage scoring system developed by Wangoo et al to describe granuloma histopathology (Wangoo et al., 2005; Johnson et al., 2006; Waters et al., 2007; Salguero et al., 2017; Palmer et al., 2021). Granulomas staged as I and II by the Wangoo system would be categorized as low-grade in this study while those granulomas classified as stage III, and IV by Wangoo et al. would be considered high-grade. Occasionally, a Wangoo stage III granuloma would fall under the low-grade designation when small foci of mineral were present with ≤10% necrosis. The Wangoo system categorizes any granuloma containing mineral as either a III or IV depending on the other characteristics present.

### Evaluation of amount of mineralization

Utilizing Von Kossa-stained slides, high- and low-grade granulomas were identified in two independent layers, at 10X magnification. Samples without identifiable lesions were excluded. High- and low-grade designations used for the H&E stains were followed to classify the lesions into their respective groups. These outlines served as the boundaries for tissue to be analyzed. A digital filter was formulated to identify calcium-containing areas representative of mineralization. Starting with algorithm Indica labs-Area quantification v2.1.7, a stain color was selected and optimized to identify mineralization. The percent area of mineralization for both high- and low-grade granulomas was calculated using the area of staining identified by the filter divided by the total area analyzed for each high- and low-grade granuloma.

### Evaluation of amount of fibrosis

Utilizing Masson’s trichrome-stained slides, high- and low-grade granulomas were outlined in two independent layers, at 10X magnification to ensure all granulomas were identified. Samples without identifiable lesions were excluded. High- and low-grade designations used for the H&E stains were followed to classify the lesions into their respective groups. A digital filter was formulated to identify collagenous areas highlighted by the trichrome stain and applied to the entire granuloma. Starting with algorithm Indica labs-Area quantification v2.1.7, a stain color was selected and optimized to identify fibrosis based on the color of collagen found in dense collagenous bundles. The filter was applied to each annotated layer. The percent fibrosis for high- and low-grade granulomas was calculated using the area of staining identified by the filter divided by the total area analyzed for each high- and low-grade granuloma.

### Statistical analysis

All statistical analyses were performed in GraphPad Prism (Version 9.5.1). E1 and E2 were treated as independent experiments. Evaluated metrics include count, average area, percent necrosis, MNGC frequency, percent mineralization, and percent fibrosis for the mediastinal and tracheobronchial lymph nodes for each animal. Mann-Whitney tests were performed for all metrics listed above to compare vaccinates and nonvaccinates across grades. Additionally, for each metric, differences between vaccination group and high- and low-grade granulomas were evaluated using a two-way ANOVA and Tukey’s multiple comparison test. For all, significance was determined when *p*-value ≤0.05. Error bars represent standard errors.

Graph construction was performed using GraphPad Prism 9 (GraphPad Software Inc., San Diego, CA). Values for the mediastinal and tracheobronchial lymph nodes for each animal were combined to yield a single data point per animal. For all analyses performed except in the evaluation of the total number of granulomas, animals without granulomas in either lymph node were excluded. In cases where an animal lacked either high- or low-grade granulomas (and therefore their calculated values were 0), these data points were excluded with exception of granuloma count.

## Results

### Granuloma number and breakdown

*BCG vaccinates had fewer granulomas compared to non-vaccinates. The ratios of high- and low-grade granulomas were similar between vaccinates and non-vaccinates.*

**Figure 2:**
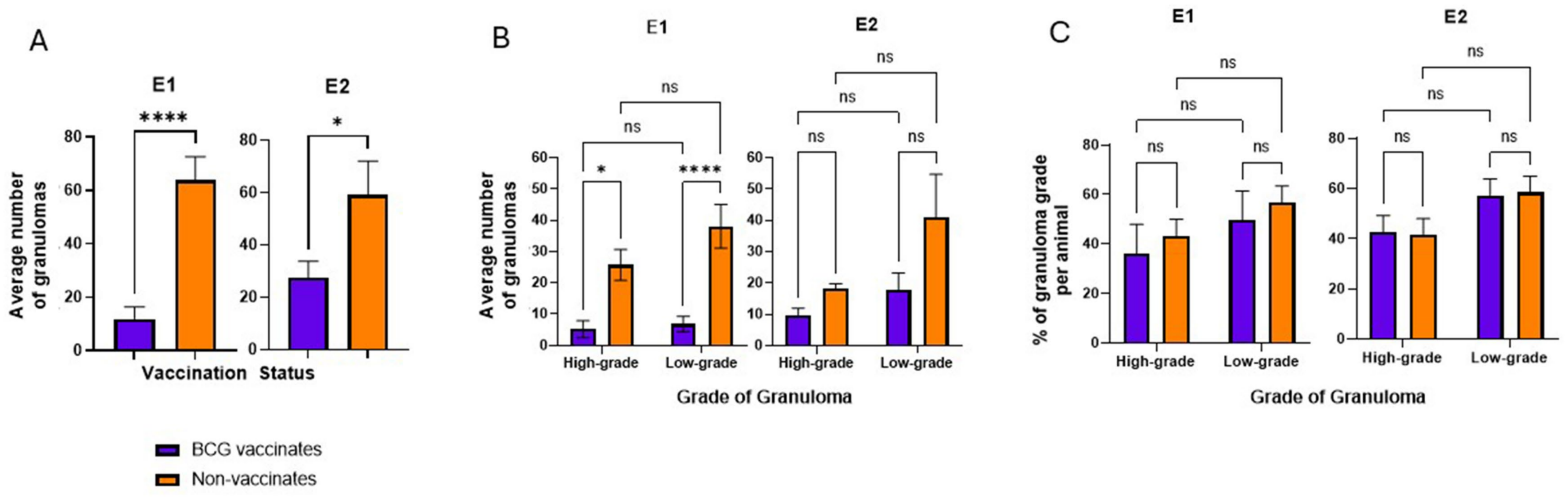
Average numbers of granulomas in BCG-vaccinated and non-vaccinated cattle infected with *M. bovis* and percentage of each granuloma grade. **(A)** the average number of granulomas per animal present in vaccinates and non-vaccinates, respectively. **(B)** the average number of granulomas present in vaccinates and non-vaccinates when separated by grade. the relative percentages of high- and low-grade granulomas. Values are presented as means ± SEM. The (* = *p* value 0.0128, * = *p* value 0.0229, **** = *p* value <0.0001, ns=not significant).

BCG vaccinates in E1 and E2 had significantly fewer granulomas than non-vaccinates (*p* < 0.0001 and *p* = 0.0229, respectively) (Figure 2A). Overall, in E1 there were 82 low-grade and 62 high-grade granulomas in BCG vaccinates and 419 lowgrade and 283 high-grade granulomas in non-vaccinates. In E2 there were 176 low-grade and 98 high-grade granulomas in BCG vaccinates and 452 low-grade and 199 high-grade granulomas in non-vaccinates. In E1, non-vaccinated animals had higher numbers of high- and low-grade granulomas compared to BCG-vaccinates (*p* = 0.013 and <0.0001 respectively) (Figure 2B). In E2 while non-vaccinates had both more low- and high-grade granulomas, this was not significant (p > 0.05). Overall, the mean number of individual granulomas per animal in E1 BCG-vaccinates was 11.9 (range 0–49) while the mean number of granulomas per animal in non-vaccinates was 63.8 (range 17–126). The mean number of individual granulomas per animal in E2 BCG-vaccinates was 27.4 (range 3–64) while the mean number of granulomas per animal in non-vaccinates was 59.2 (range 23–178). Eight lymph node samples from BCG-vaccinates lacked identifiable granulomas, and these samples were excluded for the remainder of the study.

While the number of granulomas varied markedly between BCG-vaccinates and non-vaccinates, the relative percentages of each grade of granuloma in a given animal were not significantly different (Figure 2C). There were similar percentages of high- and low- grade granulomas, within each vaccinate group. Of the total granulomas present in E1 BCG vaccinates, 57% of them were low grade while 43% were high grade. In E1 non-vaccinates these percentages were 60% and 40% respectively. In E2, low-grade and high-grade granulomas accounted for 64% and 36% respectively while non-vaccinates had 69% and 31% each of low- and highgrade lesions.

Overall, these data demonstrate BCG vaccination reduces the number of granulomas in animals challenged with virulent *M. bovis*, however the relative ratios of high- and low-grade granulomas are similar, independent of vaccination status.

### Average granuloma size and amount of necrosis

*BCG vaccination reduces the percentage of necrosis within lesions.*

**Figure 3:**
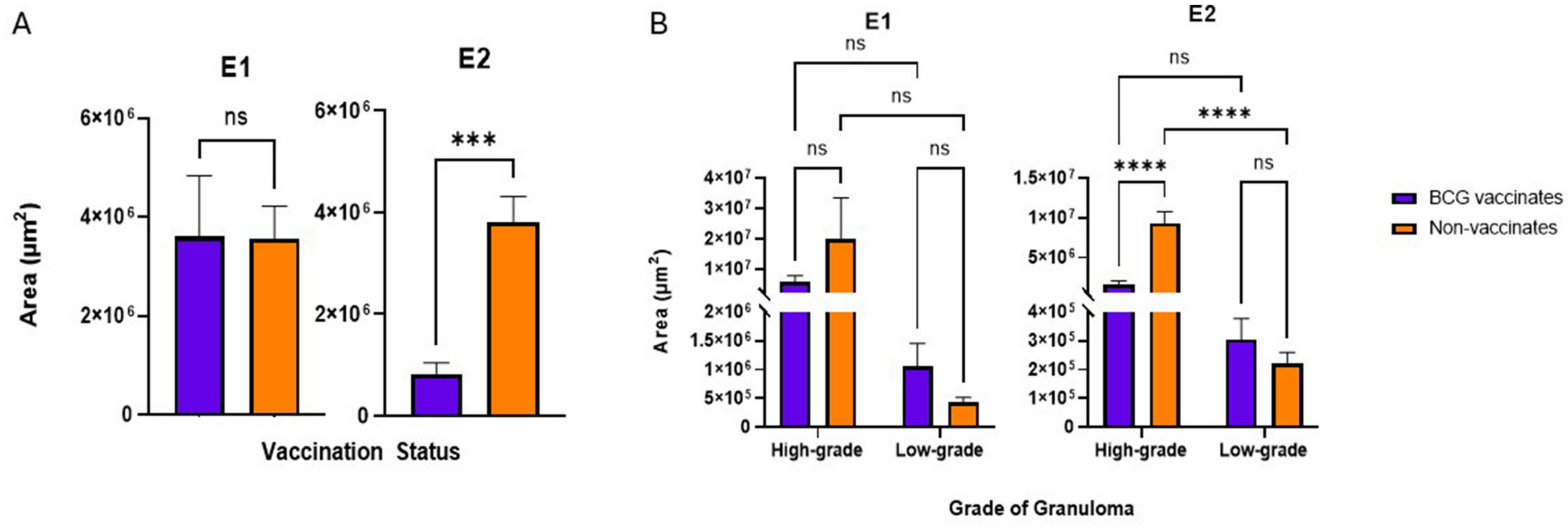
**(A)** Granuloma size by area (µm^2^) based on vaccination status. The average area of a granuloma was tabulated by dividing the total area occupied by all granulomas in the mediastinal and tracheobronchial lymph nodes of an animal by the total number of granulomas present. **(B)** Granuloma size by area (µm^2^) when broken down by grade (high or low). Values are presented as means ± SEM. (*** = *p* value 0.0004, **** = *p* value <0.0001, ns = not significant).

In E1 there were no significant differences in the average size of granulomas between vaccinates and non-vaccinates, when total granulomas were compared (Figure 3A) or when broken down by grade (Figure 3B). In contrast, when granulomas from E2 were compared, vaccinated animals had significantly smaller granulomas (*p* = 0.0006) as compared to non-vaccinated animals (Figure 3A). This difference was the result of high-grade lesions being significantly smaller in vaccinated animals (*p* < 0.0001) as compared to non-vaccinates. Low-grade granuloma size did not differ between vaccinated and non-vaccinated animals in E2.

**Figure 4:**
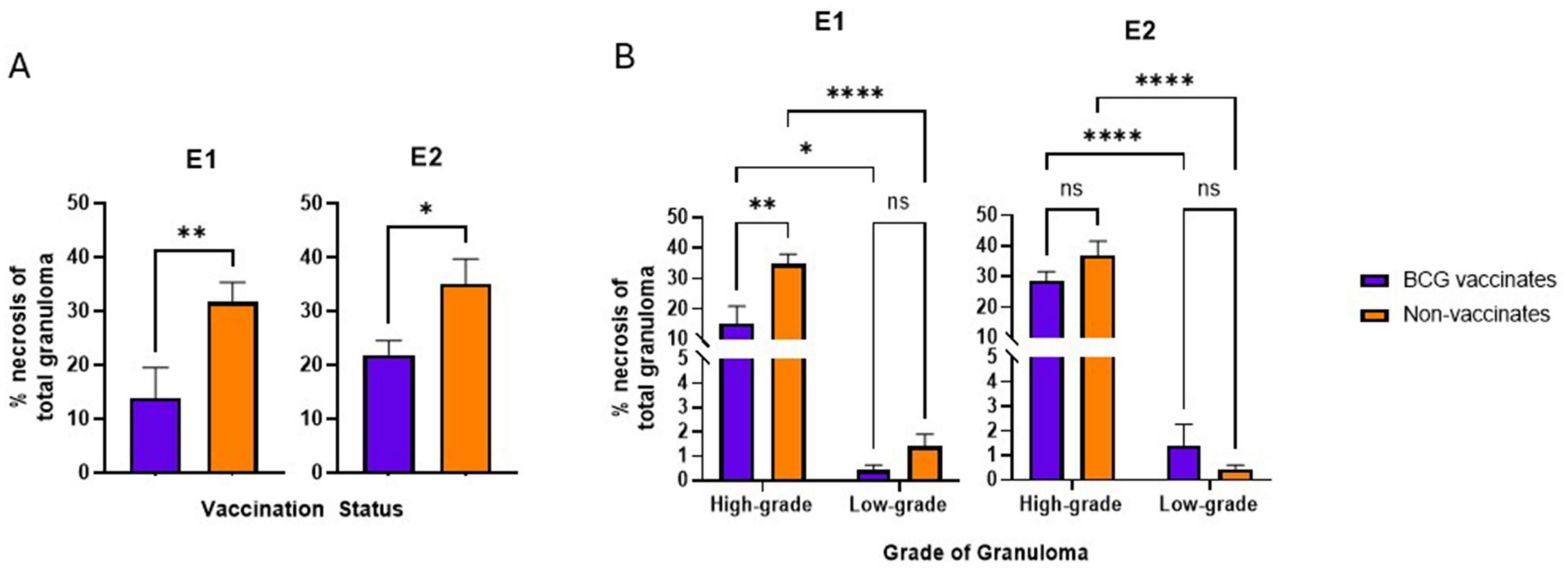
Values are broken down by vaccination group **(A)** and by vaccination group and grade **(B)**. Values are presented as means ± SEM. (* = *p* value 0.0197 for A, * = *p* value 0.0143 for B, ** = *p* value 0.0081 for A, ** = *p* value 0.0010 for B, **** = *p* value <0.0001, ns = not significant).

In this study, to be considered low-grade, the amount of necrosis had to be ≤10% the total area. However, high grade granulomas may have ≤10% of necrosis if they were multicentric or coalescing. In both E1 and E2, granulomas from vaccinates had less necrosis than non-vaccinates (*p* = 0.0081 and *p* = 0.0197 respectively) (Figure 4A). Specifically, in E1 high-grade granulomas of vaccinates had less necrosis than high-grade granulomas of non-vaccinates (*p* = 0.0010) (Figure 4B). As expected, highgrade granulomas had more necrosis than low-grade granulomas regardless of vaccination status or experimental group (*p* = 0.0143 for E1 BCG-vaccinates, *p* < 0.0001 for E1 non-vaccinates, *p* < 0.0001 for both E2 vaccinates and non-vaccinates) (Figure 4B). There were no significant differences in the percentage of necrosis within low-grade granulomas of vaccinates compared to nonvaccinates in either E1 or E2 (Figure 4B).

### Multinucleated giant cell numbers

*BCG vaccinates in E1 had fewer MNG Cover all however there were no significant differences in E2 or when comparing different granuloma grades.*

**Figure 5:**
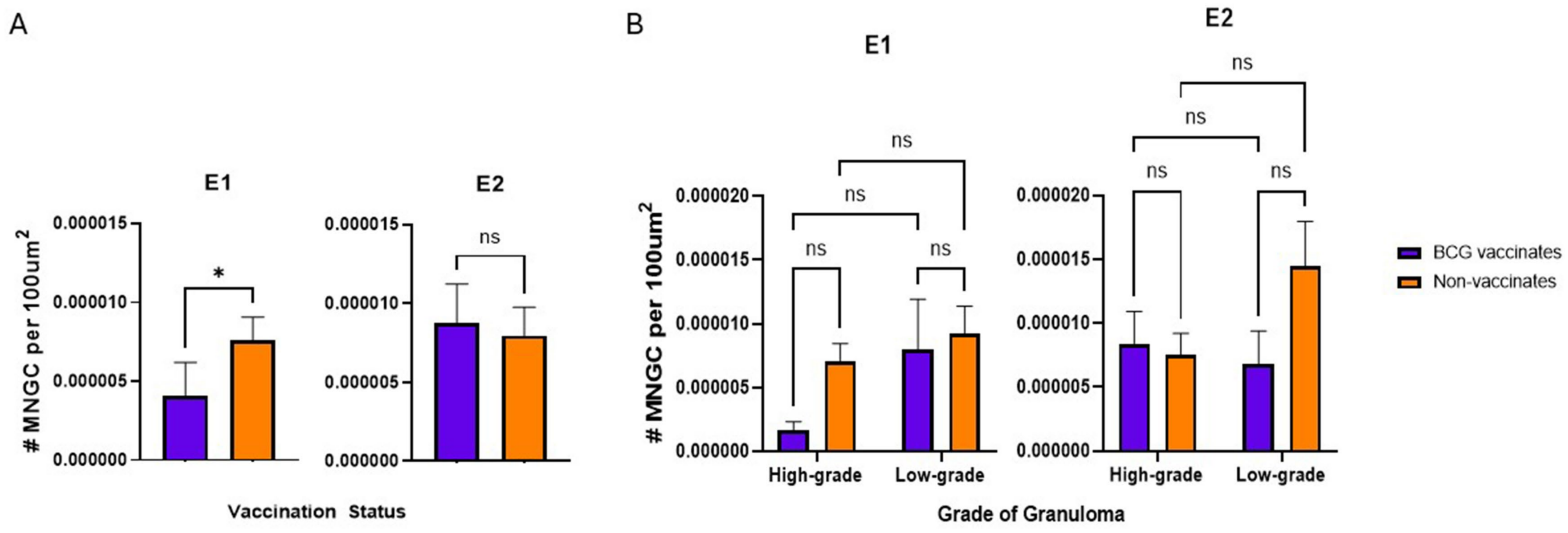
Values represent the number per 100 µm^2^ and are broken down by vaccination group **(A)** and by vaccination group and grade **(B)**. Values are presented as means ± SEM. (* = *p* value 0.04, ns = not significant).

In E1, granulomas from BCG vaccinates had fewer per 100 μm^2^ than granulomas from non-vaccinates (*p* = 0.0357) (Figure 5A). However, in E2, we did not observe any differences (*p* = 0.8633) in the number of MNGCs between granulomas from vaccinates and non-vaccinates (Figure 5A). Interestingly, when evaluated by granuloma grade, all significance was lost; there were no significant differences in the number of MNGC between high- and low-grade granulomas in either E1 or E2 (Figure 5B).

### Fibrosis and mineralization

*BCG vaccination did not alter the levels of fibrosis or mineralization within granulomas. Mineralization generally increased with the severity of the lesion*.

**Figure 6:**
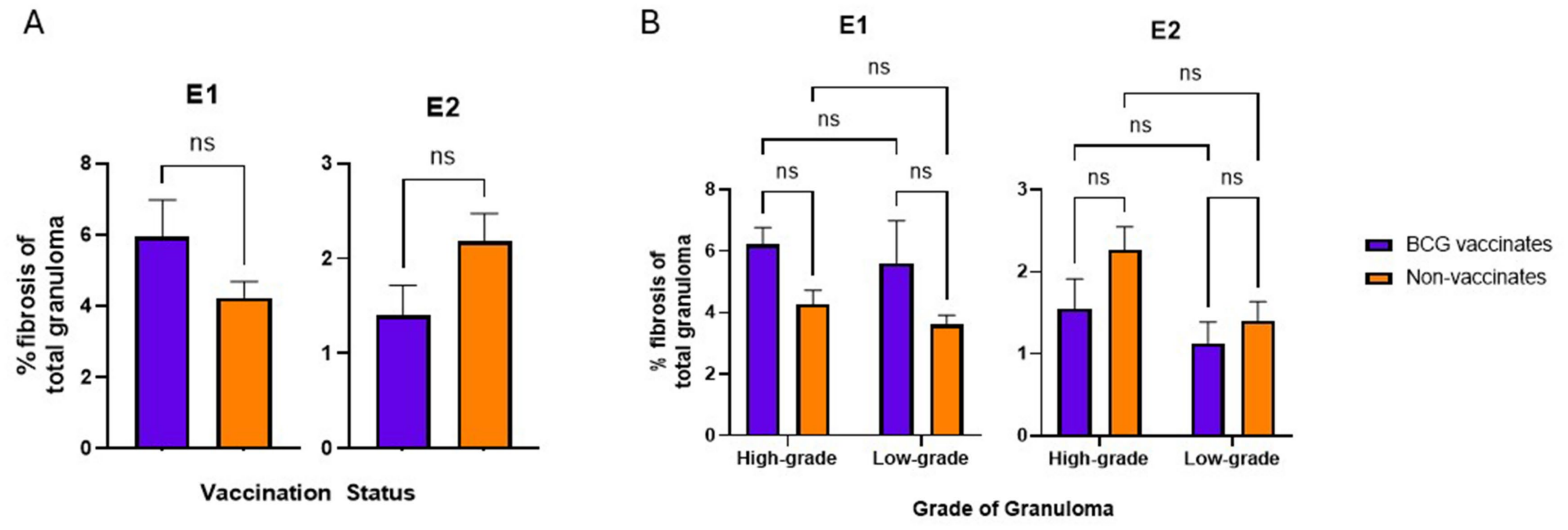
Values are broken down by vaccination group **(A)** and by vaccination group and grade **(B)**. Values are presented as means ± SEM. (ns = not significant).

**Figure 7:**
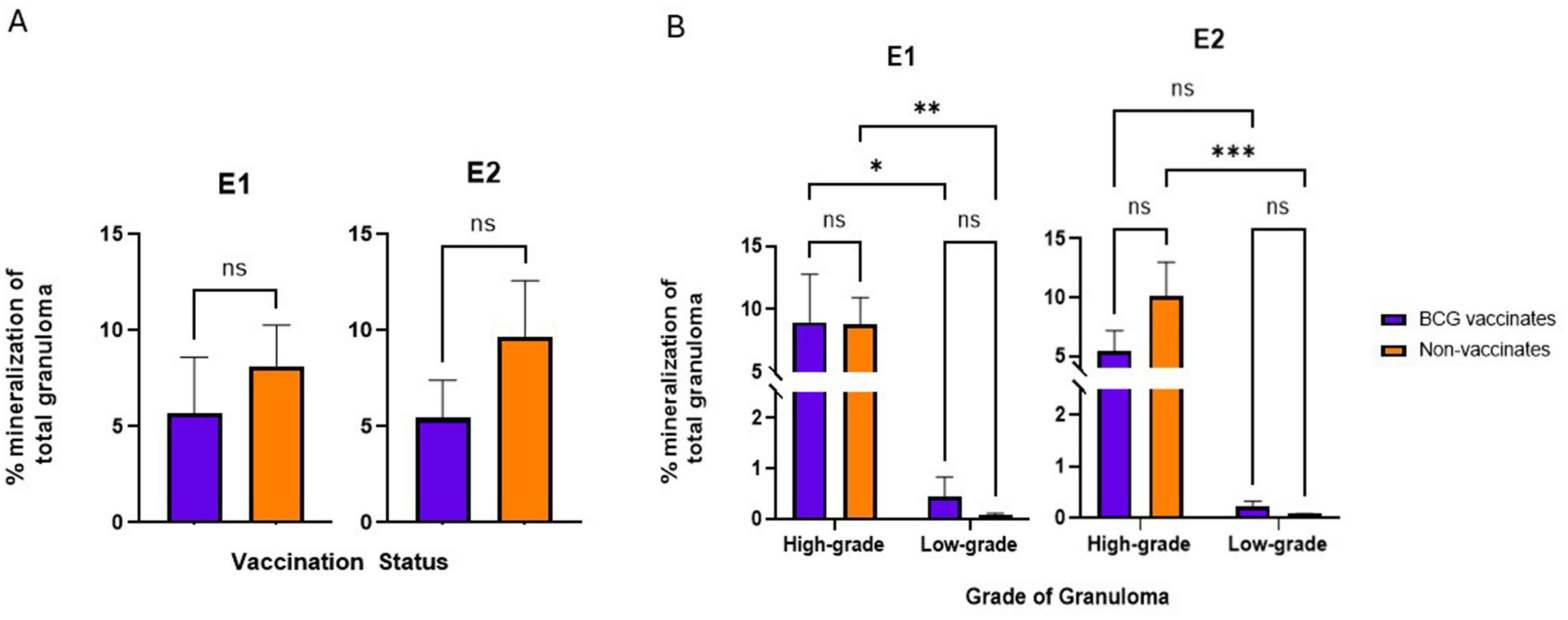
Values are broken down by vaccination group **(A)** and by vaccination group and grade **(B)**. Each dot represents one animal and its mediastinal and tracheobronchial lymph nodes. Values are presented as means ± SEM. (* = *p* value 0.04, ** = *p* value 0.008, *** = *p* value 0.0006, ns = not significant).

There were no significant differences in the percentage of fibrosis per granuloma between vaccinates and non-vaccinates for either experimental group (Figure 6A). This was also true when fibrosis was analyzed by grade (Figure 6B). When mineralization was evaluated, no significant differences were observed in the percentage of mineralization in granulomas from vaccinates and non-vaccinates (Figure 7A) and mineralization was similar between vaccinates and non-vaccinates when evaluating the same grade of granuloma (Figure 7B). In E1, granulomas from vaccinates and non-vaccinates had significantly more mineralization in the highgrade granulomas compared to the low-grade granulomas (*p* = 0.0396 for vaccinates and *p* = 0.0076 for non-vaccinates). In contrast, in E2 only granulomas from non-vaccinates had highgrade granulomas with significantly higher mineralization than low-grade lesions (*p* = 0.0006).

## Discussion

This study examined 418 granulomas from BCG-vaccinates (144 from E1 and 274 from E2) and 1,353 from non-vaccinates (702 from E1 and 651 from E2). We looked at the size of the granuloma, amount of necrosis, number of multinucleated giant cells, the amount of fibrosis and the amount of mineralization.

Previous studies which have focused on granuloma histopathology have utilized a 4-stage scoring system created by Wangoo et al (Wangoo et al., 2005; Johnson et al., 2006). While originally created to be a consistent, easy way to describe the severity of granulomas, we found it subjective in some regards, ([ex] “minimal necrotic areas”) and inappropriately absolute in others, ([ex] mineralization not mentioned until stage III or above). These aspects could make it difficult to accurately compare results from different vaccine studies from different labs. As it pertains to vaccine efficacy in tuberculosis research, the primary concern is if disease severity is reduced. While the 4-stage system is more descriptive, in this study histopathologic lesion staging was streamlined and simplified into high- and low grades based primarily on amount of necrosis. Here, lesions were categorized into either low grade, with ≤10% necrosis and high grade, with >10% necrosis and/or coalescing lesions. This binary approach allowed for pertinent, precise evaluation while eliminating some of the subjective aspects of 4 stage scoring. While any amount of necrosis could have been selected for the high and low cutoff values, we found this new scoring system to align most closely to the Wangoo system while removing subjectivity. In this study, stage I and II lesions would be considered low grade while stage III and stage IV lesions would have been classified as high grade. Granulomas designated as low grade represent those in which cellular destruction is minimal, therefore they may still be considered under host control. In contrast, granulomas designated as high grade may be interpreted as a loss of host control as necrotic cellular accumulations and/or multicentricity are features which likely benefit the bacterium and represent irreversible tissue damage (Basaraba, 2008; Basaraba and Orme, 2011). In human tuberculosis, reactivation of latent disease is associated with increased necrosis within granulomas (Russell et al., 2010). This binary microscopic scoring system is user-friendly and can be combined with gross lesion scoring if the purpose of a given study is to evaluate vaccine efficacy. The speed at which it can be applied additionally lends itself to studies utilizing large numbers of animals.

BCG decreased the lesion burden in both groups, which is in accordance to previous studies (Buddle et al., 1995; Johnson et al., 2006; Canto Alarcon et al., 2013; Dean et al., 2014; Salguero et al., 2017; Palmer et al., 2022). When evaluated as a whole, E1 vaccinated animals had an average of 11.9 granulomas per tissue section while non-vaccinated animals averaged 63.8. In E2, vaccinated animals averaged 27.4 granulomas per tissue section while nonvaccinates averaged 59.2. Interestingly, the relative breakdown of high- and low-grade granulomas did not significantly vary, with vaccinates and non-vaccinates having similar percentages of each. This suggests that the protective nature of BCG is, in part, by preventing the establishment of granulomas as opposed to necessarily preventing their progression to a higher grade. These findings are consistent with Johnson et al. and studies performed in white-tailed deer which report all stages of granulomas in lymph nodes of BCG vaccinates (Johnson et al., 2006; Nol et al., 2008; Palmer et al., 2009).

Although BCG did not prevent high grade granulomas from being formed, in E2 animals it maintained smaller lesions on average and, more specifically, within the high-grade granulomas. In E1 there were no significant differences in average granuloma area, reflecting an aspect of the variability seen within BCG vaccinates. In both E1 and E2, high-grade granulomas averaged larger areas than low-grade granulomas, irrespective of vaccination status. In both E1 and E2, vaccinates had less necrosis within lesions than non-vaccinates. In E1high-grade granulomas of nonvaccinates had more necrosis than vaccinates. This finding is reasonable, as BCG primes the immune system for a strong cell-mediated immune response, which is believed to reduce the growth of lesions (Watanabe et al., 2006; Domingo et al., 2014). Although not quantified, “satellite” granulomas, lesions closely associated with another granuloma, were present in both vaccinates and non-vaccinates. In this study, “satellite” lesions were of both high- and low-grades, a finding in contrast to previous studies which identified only low-grade granulomas (Palmer et al., 2007; Canal et al., 2017). While differences in study design between the previous studies and the current could potentially account for the disparity in satellite granuloma grades, it does raise a curious topic to scientifically explore. It is generally believed that satellite lesions represent dissemination from the nearby “primary granuloma.” Quantifying and histologically characterizing the differences between “satellite” granulomas between vaccinates and non-vaccinates may provide insight on if a vaccine reduces dissemination of bTB within the host, and provide another metric in which to measure efficacy.

While fibrosis has previously been considered a sign of an advanced or progressing lesion, our findings support data derived from simulated non-human primate granulomas which demonstrated fibrosis onset in lesions as early as ~10 days post infection or as late as after 200 days (Wangoo et al., 2005; Russell et al., 2009; Warsinske et al., 2017a; Palmer et al., 2021). In humans and non-human primate models, organized peripheral and central fibrosis has been associated with healing (Basaraba, 2008; Flynn, 2011; Palmer et al., 2022). These studies frequently involve post-chemotherapeutic treatment which is not routinely performed in cattle due to cost and risk of antibiotic residues in meat and milk, and the histopathologic composition of a healing bovine granuloma is therefore definitively unknown. Surprisingly, there were no significant differences in the percentage of fibrosis within granulomas between vaccinates or non-vaccinates, regardless of granuloma grade. This is a finding in contrast to other studies (Johnson et al., 2006; Salguero et al., 2017).

And, “Our findings show BCG does not impart protection by increasing fibrosis. Further studies evaluating the amount of central fibrosis compared to peripheral fibrosis may identify effects of BCG.

**Figure 8:**
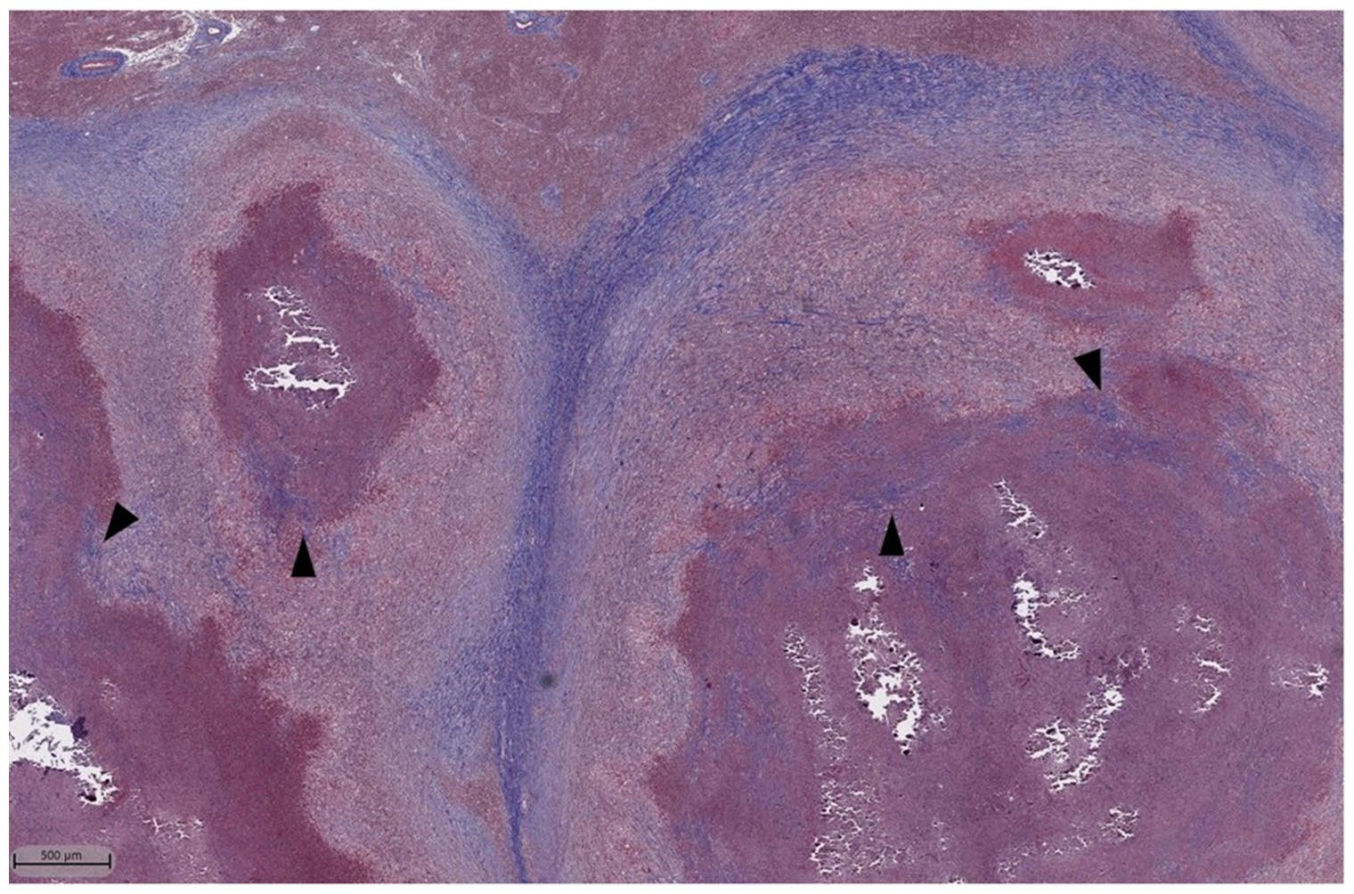
Masson’s Trichrome stain of lymph node granulomas of cattle infected with *M. bovis*. Dark blue staining represents collagen which is found prominently surrounding the granulomas. Additionally, there are areas of centralized fibrosis throughout and immediately next to areas of necrosis (arrowheads).

Dystrophic mineralization is a common sequela to necrosis and therefore it is not surprising to see levels of mineralization increased in high grade granulomas of both E1 and E2, independent of vaccination status. Nevertheless, it is interesting to note that the overall percentage of mineralization between vaccinates and nonvaccinates was non-significant. Also of note is that small amounts of mineralization were present in the low-grade granulomas. While the Wangoo categorization system attributes mineralization with increased lesion score, and therefore, increased disease severity, studies in humans and non-human primates associate mineralization with healing, similar to fibrosis (Wangoo et al., 2005; Basaraba, 2008; Gideon et al., 2022). Indeed calcification is preferable over necrosis as necrosis can harbor viable bacteria while mineralization does not (Basaraba, 2008). Our findings of small amounts of mineral in low-grade granulomas and similar overall amounts between vaccinates and non-vaccinates, puts into question how disease severity should be defined in BCG vaccination studies in cattle.

Multinucleated giant cells are associated with persistent antigen in multiple diseases (Gupta et al., 2014; Gharun et al., 2017; Pagan and Ramakrishnan, 2018; Trout and Holian, 2020; Palmer et al., 2022). We hypothesized that MNGCs would be lower in BCG vaccinates as vaccination is typically associated with lower bacterial burden and antigen persistence(Buddle et al., 2003; Hope et al., 2005; Canto Alarcon et al., 2013; Sirak et al., 2021). Additionally, we expected to see higher numbers in high-grade granulomas as severe lesions have previously been associated with higher MNGC numbers (Menin et al., 2013). Unfortunately, in both E1 and E2 we found no significant differences between granuloma grades regardless of vaccination. Our findings differ from those of Johnson et al, which reported a significant reduction in the numbers of MNGCs in BCG vaccinated animals (Johnson et al., 2006). This difference could be due to the fact that Johnson et al, included only Langhan’s type giant cells. Although not explicitly quantified, and without a defined description of MNGC requirements, Salguero et al additionally reported a decrease in the number of MNGCs in BGC-vaccinated cattle (Salguero et al., 2017). Frequently the Langhan’s-type MNGC, with its nuclei arranged in a horseshoe shape, is considered the hallmark of tuberculosis, but in-fact, multiple other types are present and transformation from one morphology to another has been documented in cell culture (Singhal et al., 2011; Palmer et al., 2016; Pagan and Ramakrishnan, 2018). In the current study, we identified any cell containing three nuclei within a continuous cytoplasm as a multinucleated giant cell, regardless of morphology. These findings in contrast to other studies suggests this is a metric which warrants further investigation into type, function and to whom (host or microbe) this unique cell type benefits.

One limitation of this study was in interpreting coalescing lesions which had effaced the majority of the lymph node parenchyma. These cases arose occasionally in both vaccinates and non-vaccinates. This phenomenon is well document in lymph nodes of chronically infected cattle, where it may completely erase any semblance of a normal lymph node (Domingo et al., 2014). BCG provides variable protection, and animals with such severely affected lymph nodes may represent animals which failed to respond appropriately to the vaccine. It would be interesting to investigate differences present in these vaccine-non-responders and compare granuloma level cytokine and chemokine differences. As it was difficult to determine exactly how many granulomas coalesced, these lesions were counted as single, large granulomas. While minimally decreasing total granuloma numbers, these cases could dramatically alter average granulomas sizes.

Although not the objective of this study, the use of banked samples from two separate experiments has highlighted an important aspect of BCG and M. bovis research. While we expected both experimental groups to behave similarly, in fact there were 3 parameters where only one experimental group had significance between BCG vaccinates and non-vaccinates (granuloma and MNGC numbers in E1 and average area in E2). Experimental groups varied based on age of vaccination, dose of BCG and strain and dose of challenge. It would be helpful to directly compare each of these variables to determine how each affects resulting lesions.

And, “Nevertheless, these authors suggest vaccinating and challenging animals the same day or utilizing the same seed stocks to eliminate these variabilities.” As a result, simple optical density (OD) readings are not reliable, and due to the lengthy culture time, exact dosages are often determined retrospectively. Although the dosages between groups were not exactly the same, they were well within the range of numerous other BCG efficacy studies conducted by us and other investigators (Hope et al., 2005; Waters et al., 2007; Vordermeier et al., 2009).

Though not investigated in this study, it would be interesting to measure bacterial load between high- and low-grade granulomas between vaccination groups and how this may correlate with the metrics measured. While Ziehl-Neelson (ZN) staining for mycobacteria has historically been performed in histopathologic examinations of bTB it was not performed in this study due to limitations of the software and the relative insensitivity of ZN staining in formalin-fixed, paraffin-embedded tissues (Fukunaga et al., 2002). As quantitative culture would be impossible due to the small size of some of the lesions, techniques such as RNA *in-situ* hybridization, which would allow on-slide examination should be considered.

Our study provides further support to show that BCG vaccination reduced granuloma formation within the pulmonary lymph nodes of cattle following aerosol challenge with virulent *M. bovis*. Once established, BCG does not prevent the development of severe lesions, and the relative breakdown of high- and low- grade granulomas is similar to that of non-vaccinates. BCG may reduce tissue destruction as not only did E2 vaccinate granulomas have a smaller average size, but necrosis was reduced in granulomas of both E1 and E2 vaccinates. The role of BCG vaccination in altering MNGC density is unclear as numbers were reduced in E1, but significance was lost when evaluating by grade. Finally, fibrosis and mineralization were not altered by BCG vaccination in either experimental group suggesting these may be vaccine-independent events.

## Data availability statement

The original contributions presented in the study are included in the article, further inquiries can be directed to the corresponding author.

## Ethics statement

The animal study was reviewed and approved by Institutional Animal Care and Use Committee at National Animal Disease Center.

## Author contributions

CK, PB, and MP: experiment design. CK and MP: sample collection and experiments. CK and EP: data analysis. CK: manuscript preparation. CK, PB, EP, and MP: manuscript editing. All authors contributed to the article and approved the submitted version.

## Funding

Financial support for these studies were provided by the United States Department of Agriculture, Agricultural Research Service Project (CRIS #5030–32000-222). This research did not receive any specific grant from funding agencies in the public, commercial, or not-for-profit sectors.

## Authors disclaimer

USDA is an equal opportunity provider and employer. Mention of trade names or commercial products in this publication is solely for the purpose of providing specific information and does not imply recommendation or endorsement by the U.S. Department of Agriculture.

## Conflict of interest

The authors declare that the research was conducted in the absence of any commercial or financial relationships that could be construed as a potential conflict of interest.

## Publisher’s note

All claims expressed in this article are solely those of the authors and do not necessarily represent those of their affiliated organizations, or those of the publisher, the editors and the reviewers. Any product that may be evaluated in this article, or claim that may be made by its manufacturer, is not guaranteed or endorsed by the publisher.
