# Corrigendum: Histopathologic differences in granulomas of *Mycobacterium bovis* bacille Calmette Guérin (BCG) vaccinated and non-vaccinated cattle with bovine tuberculosis

**DOI:** 10.3389/fmicb.2025.1571061

**Published:** 2025-03-27

**Authors:** C. Kanipe, P. M. Boggiatto, E. J. Putz, M. V. Palmer

**Affiliations:** ^1^Infectious Bacterial Diseases Research Unit, National Animal Disease Center, Agricultural Research Service (USDA), Ames, IA, United States; ^2^Immunobiology Graduate Program, Iowa State University, Ames, IA, United States

**Keywords:** granuloma, *Mycobacterium bovis*, BCG, bovine tuberculosis, tuberculosis vaccine

In the published article, there was an error in the legend for Figure 2 as published. The original legend stated, “Absolute numbers of granulomas in BCG-vaccinated and non-vaccinated cattle infected with *M. bovis* and percentage of each granuloma grade. **(A)** the total number of granulomas present in vaccinates and non-vaccinates, respectively. Each dot represents one animal and the combined total of granulomas in its mediastinal and tracheobronchial lymph nodes. **(B)** the relative percentages of high- and low-grade granulomas. Values are presented as means ± SEM. The (^****^=*p* value < 0.0001).”

The corrected legend appears below.

**Figure 2**. Average numbers of granulomas in BCG-vaccinated and non-vaccinated cattle infected with *M. bovis* and percentage of each granuloma grade. **(A)** The average number of granulomas per animal present in vaccinates and non-vaccinates, respectively. **(B)** The average number of granulomas present in vaccinates and non-vaccinates when separated by grade. The relative percentages of high- and low-grade granulomas. Values are presented as means ± SEM. The (*= *p* value 0.0128, * = *p* value 0.0229, **** = *p* value < 0.0001, ns, not significant).

**Figure 2:**
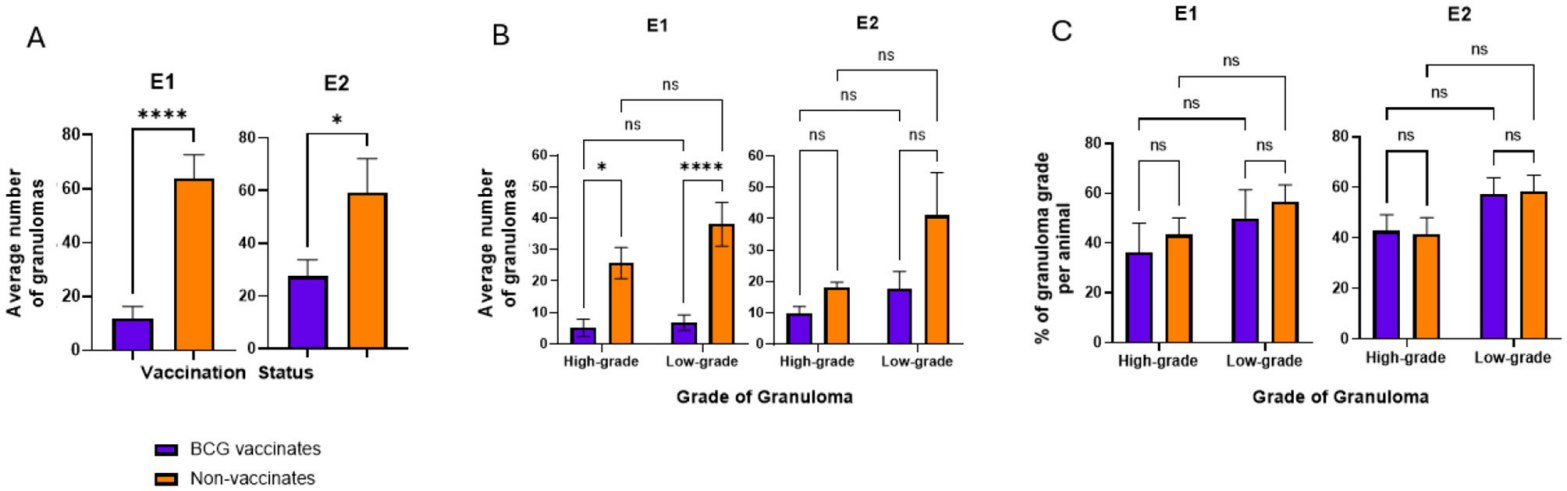


In the published article, there was an error in the legend for Figure 3 as published. The original legend stated incorrect *p* values “(^*^=*p* value 0.0434, ^**^= *p* value 0.0064, ns=not significant).”

The corrected legend appears below.

**Figure 3. (A)** Granuloma size by area (μm^2^) based on vaccination status. The average area of a granuloma was tabulated by dividing the total area occupied by all granulomas in the mediastinal and tracheobronchial lymph nodes of an animal by the total number of granulomas present. **(B)** Granuloma size by area (μm^2^) when broken down by grade (high or low). Values are presented as means ± SEM (^***^ = *p* value 0.0004, ^****^ = *p* value < 0.0001, ns, not significant).

**Figure 3:**
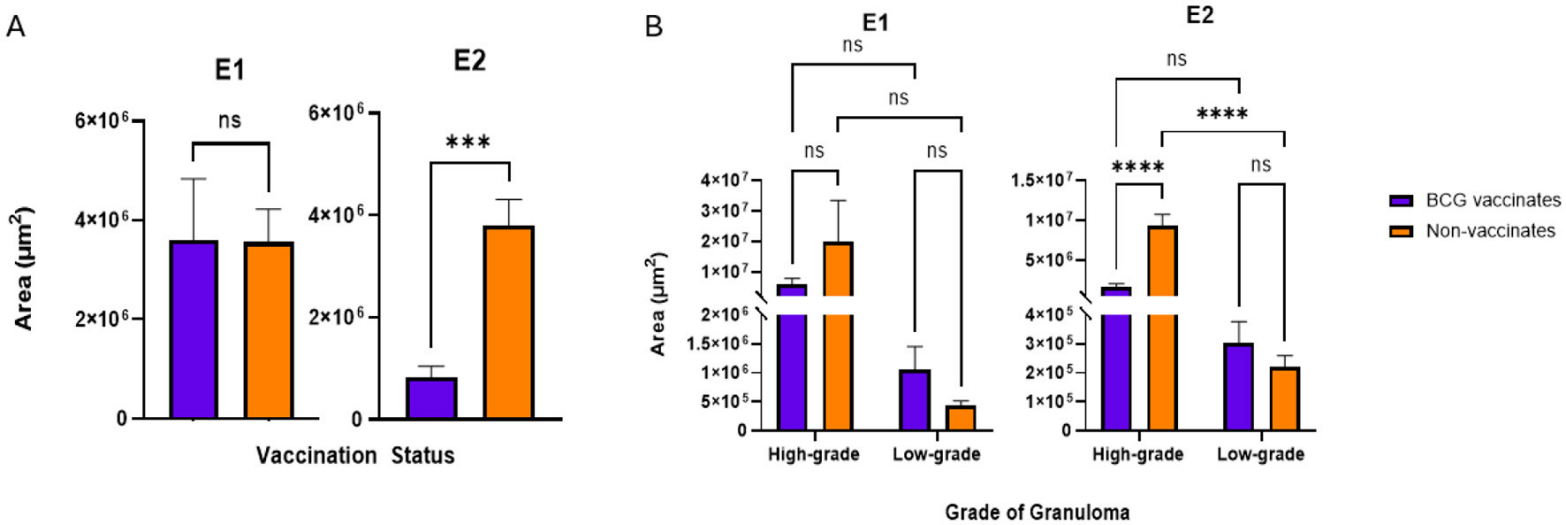


In the published article, there was an error in the legend for Figure 4 as published. The original legend stated incorrect *p* value s, “ (^**^=*p* value 0.0029 for A, ^**^= *p* value 0.0080 for B, ^****^= *p* value *p* value < 0.0001).”

The corrected legend appears below.

**Figure 4**. Values are broken down by vaccination group **(A)** and by vaccination group and grade **(B)**. Values are presented as means ± SEM. (^*^ = *p* value 0.0197 for **(A)**, ^*^ = *p* value 0.0143 for **(B)**, ^**^ = *p* value 0.0081 for **(A)**, ^**^= *p* value 0.0010 for **(B)**, ^****^ = *p* value < 0.0001, ns, not significant).

**Figure 4:**
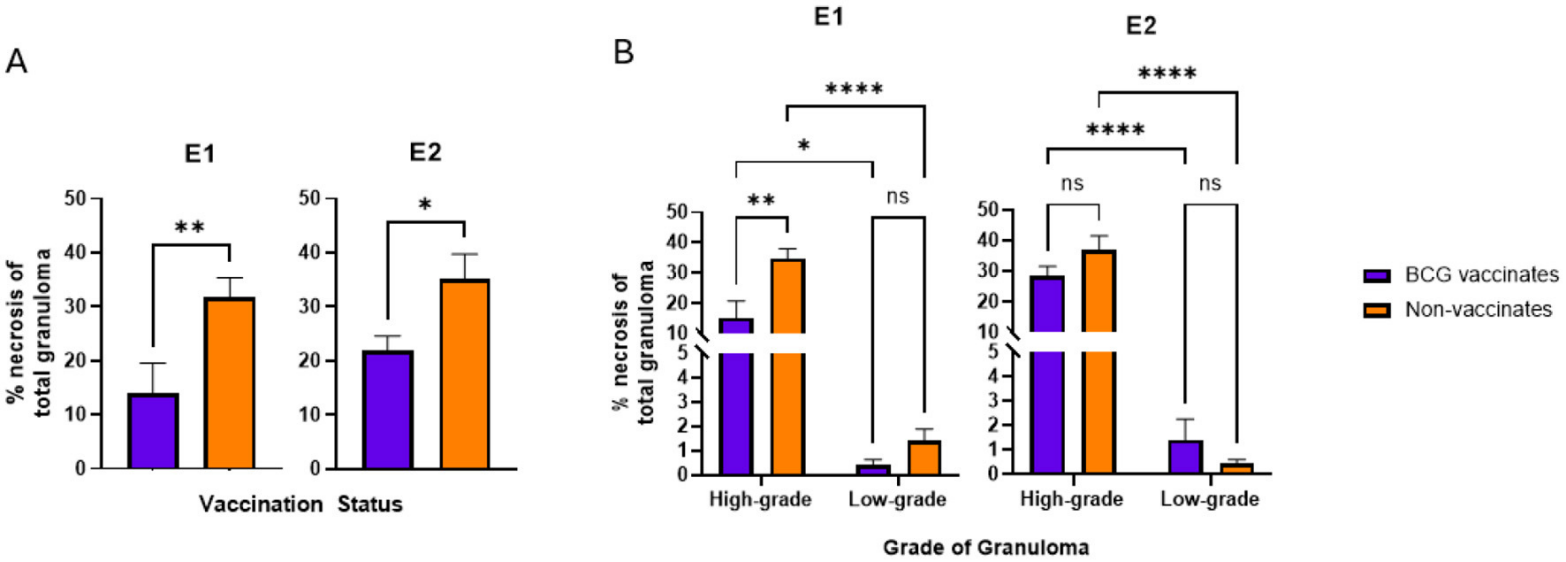


In the published article, there was an error in the legend for Figure 5 as published. The original legend stated incorrect *p* values “(ns=not significant).”

The corrected legend appears below.

**Figure 5**. Values represent the number per 100 μm^2^ and are broken down by vaccination group **(A)** and by vaccination group and grade **(B)**. Values are presented as means ± SEM (^*^ = *p* value 0.04, ns, not significant).

**Figure 5:**
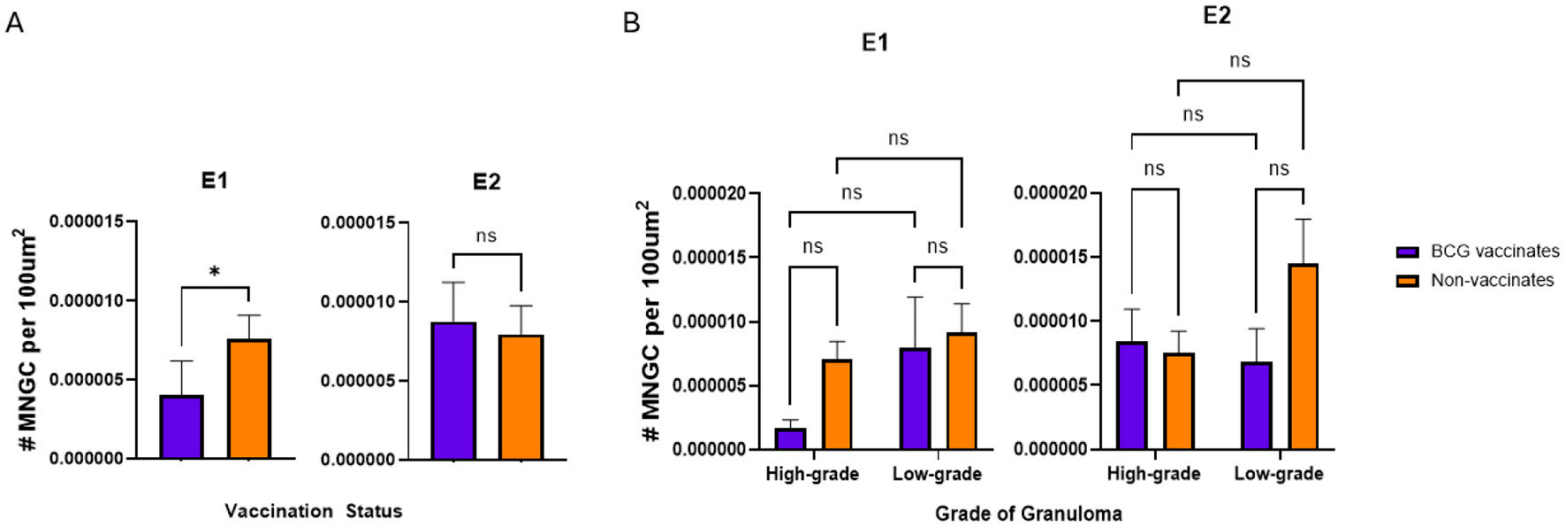


In the published article, there was an error in the legend for Figure 6 as published. The original legend stated incorrect *p* values “(^*^ = *p* value 0.0344, ns=not significant).”

The corrected legend appears below.

**Figure 6**. Values are broken down by vaccination group **(A)** and by vaccination group and grade **(B)**. Values are presented as means ± SEM (ns, not significant).

**Figure 6:**
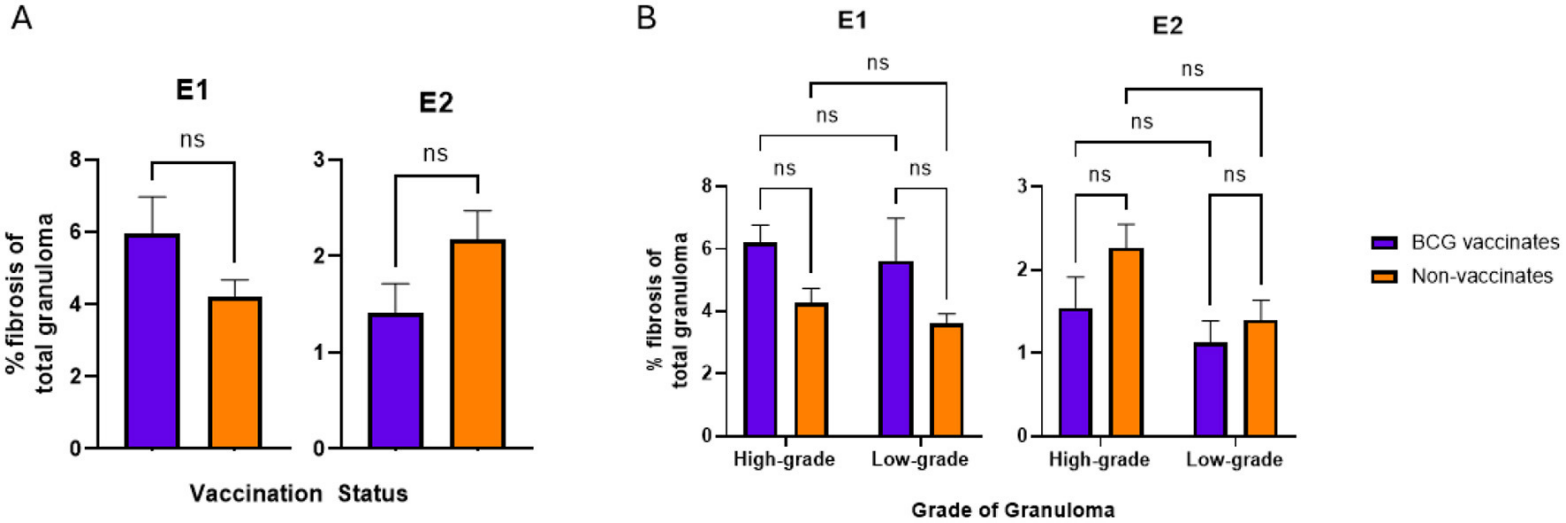


In the published article, there was an error in the legend for Figure 7 as published. The original legend stated incorrect *p* values “(^***^=*p* value 0.0009, ^****^= *p* value < 0.0001, ns=not significant).”

The corrected legend appears below.

**Figure 7**. Values are broken down by vaccination group **(A)** and by vaccination group and grade **(B)**. Each dot represents one animal and its mediastinal and tracheobronchial lymph nodes. Values are presented as means ± SEM (^*^ = *p* value 0.04, ^**^ = *p* value 0.008, ^***^ = *p* value 0.0006, ns, not significant).

**Figure 7:**
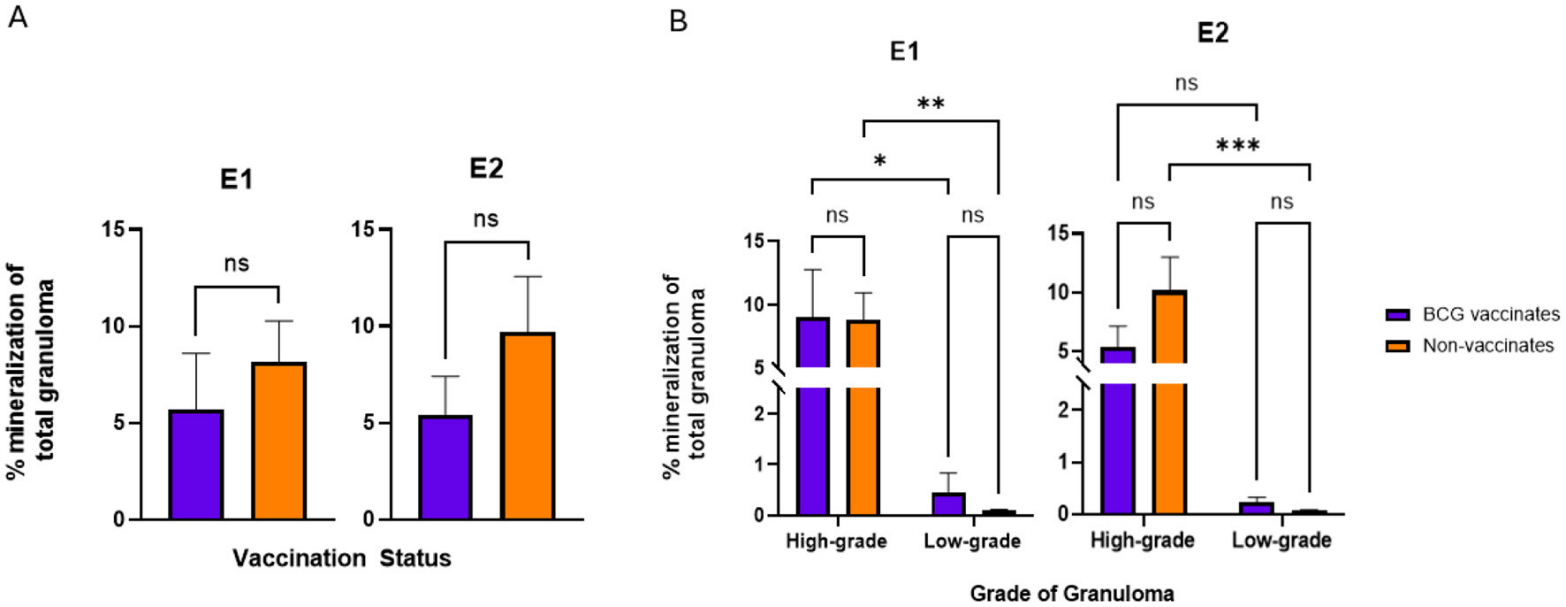


In the published article, there was an error in Figures 2–7 as published. Due to differences in challenge strains, experimental groups needed to be treated independently. The corrected figures appear below. The corrected legends are listed in the previous section.

In the published article, there was an error. In fact, two different challenge strains were used in the two experiments, not one. This resulted in the need to analyze groups separately and not combine them. This issue has caused multiple sections to be updated to reflect the correct comparisons and statistics.

A correction has been made to **Abstract**, 1. This section previously stated:

“BCG vaccinates had fewer granulomas overall and smaller high-grade granulomas with less necrosis than non-vaccinates. The relative numbers of high- and low- grade lesions were similar as were the amounts of mineralization and the density of MNGC. The amount of fibrosis was higher in low-grade granulomas from vaccinates compared to non-vaccinates. Collectively, these findings suggest that BCG vaccination reduces bacterial establishment, resulting in the formation of fewer granulomas. In granulomas that form, BCG has a protective effect by containing their size, reducing the relative amount of necrosis, and increasing fibrosis in low-grade lesions. Vaccination did not affect the amount of mineralization or density of MNGC”

The corrected sentences appear below:

“In one set of banked samples BCG vaccinates had fewer granulomas overall and lower numbers of multinucleated giant cells. In the other set of samples, lesions of vaccinates were significantly smaller. In both experimental groups vaccinates had less necrosis than non-vaccinates. The relative numbers of high- and low- grade lesions were similar between vaccinates and non-vaccinates of both groups as were the amounts of fibrosis and mineralization. Collectively, these findings demonstrate the variability of protection offered by BCG. It suggests that BCG vaccination may serve to reduce bacterial establishment, resulting in the formation of fewer granulomas and in granulomas that form, that it may have a protective effect by containing their size and reducing the relative amount of necrosis.”

A correction has been made to **Materials and Methods**, *Samples* 1. This section previously stated:

“Briefly, in Experiment 1, 23 castrated Holstein steers of 4-5 months of age were divided into two groups: non-vaccinates (*n* = 11) and BCG vaccinates (*n* = 12). Animals were kept on pasture prior to the start of the study. As environmental bacteria are present in our area, and in order to prevent possible confounding responses to BCG vaccination, animals were confirmed non-reactive to *Mycobacterium avium* (*M. avium*) via interferon gamma release assay (IGRA) immediately before the start of the study. Animals in the vaccinated group received a subcutaneous injection 1 ml of 5 X 10^5^ CFU of BCG Danish. Three months following vaccination, both vaccinates and non-vaccinates received 5.5 x 10^2^ CFU of *M. bovis* strain 1315 via aerosolization as described elsewhere (Palmer et al., 2002). Strain 1315 is a virulent field strain obtained from a white-tailed deer in Michigan, USA.

The corrected sentences appear below:

“Briefly, in Experiment 1 (E1), 23 castrated Holstein steers of 4–5 months of age were divided into two groups: non-vaccinates (*n* = 11) and BCG vaccinates (*n* = 12). Animals were kept on pasture prior to the start of the study. As environmental bacteria are present in our area, and to prevent possible confounding responses to BCG vaccination, animals were confirmed non-reactive to *Mycobacterium avium* (*M. avium*) via interferon gamma release assay (IGRA) immediately before the start of the study. Animals in the vaccinated group received a subcutaneous injection 1 ml of 5 X 10^5^ CFU of BCG Danish. Three months following vaccination, both vaccinates and non-vaccinates received 5.5 x 10^2^ CFU of *M. bovis* strain 10-7428 via aerosolization as described elsewhere (Palmer et al., 2002).”

A correction has been made to **Materials and methods**, *Samples* 1. This section previously stated:

“In Experiment 2, 21 newborn Holstein steers were divided into two groups: non-vaccinates (*n* = 11) and BCG vaccinates (*n* = 10). Animals were bottle-raised on a pasteurized milk product and kept in clean pens to reduce exposure to environmental mycobacteria. Due to the cleanliness of the housing situation the risk of exposure to environmental mycobacteria was considered low and IGRA testing for *M. avium* was not performed. Animals in the vaccinated group received a subcutaneous injection 1 ml of 1 X 10^6^ CFU of BCG Danish at 2 weeks of age. Three months following vaccination, both vaccinates and non-vaccinates received 1 x 10^3^ CFU of *M. bovis* strain 1315 via aerosolization.”

The corrected sentences appear below:

“In Experiment 2 (E2), 21 newborn Holstein steers were divided into two groups: non-vaccinates (*n* = 11) and BCG vaccinates (*n* = 10). Animals were bottle-raised on a pasteurized milk product and kept in clean pens to reduce exposure to environmental mycobacteria. Due to the cleanliness of the housing situation the risk of exposure to environmental mycobacteria was considered low and IGRA testing for *M. avium* was not performed. Animals in the vaccinated group received a subcutaneous injection 1 ml of 1 X 10^6^ CFU of BCG Danish at 2 weeks of age. Three months following vaccination, both vaccinates and non-vaccinates received 1 x 10^3^ CFU of *M. bovis* strain 95-1315 via aerosolization.”

A correction has been made to **Materials and methods**, *Samples* 1. This section previously stated:

“The two groups used in this study varied in age at the time of vaccination, 2 wks vs. 4–5 mos. It was previously reported that neonatally vaccinated animals had at least as robust an immune response as adults (Hope et al., 2005). Nevertheless, to account for differences between experimental groups we evaluated the effect of group within all statistical analyses and found significance only for fibrosis metrics where group was included as a fixed effect (see stats section).”

This statement has been removed.

A correction has been made to **Materials and methods**, *Statistical analysis* 9. This section previously stated.

“For all scored granuloma metrics (count, average area, percent necrosis, MNGC frequency, percent mineralization, and percent fibrosis) data were analyzed with a simple linear regression model (lm) in R (version 4.2.1). Each scored metric was evaluated independently, fitting grade, vaccination status, and their interaction as fixed effects. A fixed effect of “group,” denoting the two separate experimental cohorts, was evaluated for model fit for each metric and only deemed appropriate to include for analysis of percent fibrosis. A pairwise comparison of Least Squares means (lsmeans package) was utilized to determine significant differences between specific grade and vaccination group contrasts.”

The corrected sentences appear below:

“All statistical analyses were performed in GraphPad Prism (Version 9.5.1). E1 and E2 were treated as independent experiments. Evaluated metrics include count, average area, percent necrosis, MNGC frequency, percent mineralization, and percent fibrosis for the mediastinal and tracheobronchial lymph nodes for each animal. Mann-Whitney tests were performed for all metrics listed above to compare vaccinates and non-vaccinates across grades. Additionally, for each metric, differences between vaccination group and high- and low-grade granulomas were evaluated using a two-way ANOVA and Tukey's multiple comparison test.”

A correction has been made to **Results**, *Granuloma number and breakdown* 1. This section previously stated:

“Non-vaccinated animals had higher numbers of granulomas compared to BCG-vaccinates (p < 0.0001) (Figure 2A). The mean number of individual granulomas per animal in BCG-vaccinates was 18.95 (range 0-51) while the mean number of granulomas per animal in non-vaccinates was 61.5 (range 17-178). In total, 1770 granulomas were evaluated. BCG vaccinates accounted for 417 (23.6%) of these granulomas while 1353 (76.4%) granulomas were in lymph nodes of non-vaccinates.”

The corrected sentences appear below:

“BCG vaccinates in E1 and E2 had significantly fewer granulomas than non-vaccinates (*p* < 0.0001 and *p* = 0.0229, respectively) (Figure 2A). Overall, in E1 there were 82 low-grade and 62 high-grade granulomas in BCG vaccinates and 419 low-grade and 283 high-grade granulomas in non-vaccinates. In E2 there were 176 low-grade and 98 high-grade granulomas in BCG vaccinates and 452 low-grade and 199 high-grade granulomas in non-vaccinates. In E1, non-vaccinated animals had higher numbers of high- and low-grade granulomas compared to BCG-vaccinates (*p* = 0.013 and < 0.0001 respectively) (Figure 2B). In E2 while non-vaccinates had both more low- and high-grade granulomas, this was not significant (*p* > 0.05). Overall, the mean number of individual granulomas per animal in E1 BCG-vaccinates was 11.9 (range 0–49) while the mean number of granulomas per animal in non-vaccinates was 63.8 (range 17–126). The mean number of individual granulomas per animal in E2 BCG-vaccinates was 27.4 (range 3–64) while the mean number of granulomas per animal in non-vaccinates was 59.2 (range 23–178).”

A correction has been made to **Results**, *Granuloma number and breakdown 2*. This section previously stated:

“While the number of granulomas varied markedly between BCG-vaccinates and non-vaccinates, the relative percentages of each grade of granuloma were similar (Figure 2B). There were approximately equal numbers of high- and low- grade granulomas, within each vaccinate group. Of the total granulomas present in BCG vaccinates, 58.52% of them were low grade while 43.14% were high grade. In non-vaccinates these percentages were 57.56% and 42.44% respectively. Overall, these data indicate that there is a trend for fewer high-grade granulomas compared to low grade granulomas independent of vaccination status (p=0.551 and p=0.0627 for non-vaccinates and vaccinates, respectively).”

The corrected sentences appear below:

“While the number of granulomas varied markedly between BCG-vaccinates and non-vaccinates, the relative percentages of each grade of granuloma in a given animal were not significantly different (Figure 2C). There were similar percentages of high- and low- grade granulomas, within each vaccinate group. Of the total granulomas present in E1 BCG vaccinates, 57% of them were low grade while 43% were high grade. In E1 non-vaccinates these percentages were 60% and 40% respectively. In E2, low-grade and high-grade granulomas accounted for 64% and 36% respectively while non-vaccinates had 69% and 31% each of low- and high-grade lesions.

Overall, these data demonstrate BCG vaccination reduces the number of granulomas in animals challenged with virulent *M. bovis*, however the relative ratios of high- and low-grade granulomas are similar, independent of vaccination status.”

A correction has been made to **Results**, *Average granuloma size and amount of necrosis* 1. This section previously stated:

“- BCG vaccinates had smaller high-grade granulomas and lesions with less necrosis.

There was no significant difference (p=0.0675) in the average size of granulomas between vaccinates and non-vaccinates, however lesions from non-vaccinated animals tended to be smaller (Figure 3A). Additionally, low-grade granulomas did not significantly vary in size between vaccinates and non-vaccinates however high-grade granulomas were larger in non-vaccinates than vaccinates (p=0.0434) (Figure 3B). Within vaccination groups, BCG vaccinates had similarly sized low- and high-grade granulomas (p=0.6296) while non-vaccinates had larger high-grade granulomas compared to low-grade granulomas (p=0.0064).”

The corrected sentences appear below:

“-BCG vaccination reduces the percentage of necrosis within lesions.

In E1 there were no significant differences in the average size of granulomas between vaccinates and non-vaccinates, when total granulomas were compared (Figure 3A) or when broken down by grade (Figure 3B). In contrast, when granulomas from E2 were compared, vaccinated animals had significantly smaller granulomas (*p* = 0.0006) as compared to non-vaccinated animals (Figure 3A). This difference was the result of high-grade lesions being significantly smaller in vaccinated animals (*p* < 0.0001) as compared to non-vaccinates. Low-grade granuloma size did not differ between vaccinated and non-vaccinated animals in E2.”

A correction has been made to **Results**, *Average granuloma size and amount of necrosis* 2. This section previously stated:

“BCG vaccinates had lesions containing significantly (p=0.0029) less tissue destruction as evidenced by a lower percentage of necrosis than non-vaccinates (Figure 4A). As a result of the categorization method, necrosis was expected to be substantially higher in high grade granulomas compared to low grade and this was true for both vaccinates and non-vaccinates (p < 0.001 for both). There was no significant difference in the percentage of necrosis within low-grade granulomas of vaccinates compared to non-vaccinates with values being nearly identical (p = 0.9766) (Figure 4B). High-grade granulomas of non-vaccinates contained a significantly (p=0.0080) higher percentage of necrosis than high-grade granulomas from BCG-vaccinates.”

The corrected sentences appear below:

“In both E1 and E2, granulomas from vaccinates had less necrosis than non-vaccinates (*p* = 0.0081 and *p* = 0.0197 respectively) (Figure 4A). Specifically, in E1 high-grade granulomas of vaccinates had less necrosis than high-grade granulomas of non-vaccinates (*p* = 0.0010) (Figure 4B). As expected, high-grade granulomas had more necrosis than low-grade granulomas regardless of vaccination status or experimental group (*p* = 0.0143 for E1 BCG-vaccinates, *p* < 0.0001 for E1 non-vaccinates, *p* < 0.0001 for both E2 vaccinates and non-vaccinates) (Figure 4B). There were no significant differences in the percentage of necrosis within low-grade granulomas of vaccinates compared to non-vaccinates in either E1 or E2 (Figure 4B).”

A correction has been made to **Results**, *Multinucleated giant cell numbers* 1. This section previously stated:

“-MNGC numbers were not dependent on vaccination status or lesion severity.

No significant differences were found in the density of MNGC between vaccination groups (Figure 5A). When separated by grade, there were no significant differences in the number of MNGC between high- and low-grade granulomas, however there was a trend (p=0.0632) for low grade granulomas of non-vaccinates to contain a higher number of MNGC than high grade granulomas of the same group (Figure 5B).”

The corrected sentences appear below:

“-BCG vaccinates in E1 had fewer MNGC overall however there were no significant differences in E2 or when comparing different granuloma grades.

In E1, granulomas from BCG vaccinates had fewer per 100 μm^2^ than granulomas from non-vaccinates (*p* = 0.0357) (Figure 5A). However, in E2, we did not observe any differences (*p* = 0.8633) in the number of MNGCs between granulomas from vaccinates and non-vaccinates (Figure 5A). Interestingly, when evaluated by granuloma grade, all significance was lost; there were no significant differences in the number of MNGC between high- and low-grade granulomas in either E1 or E2 (Figure 5B).”

A correction has been made to **Results**, *Fibrosis and Mineralization* 1. This section previously stated:

“-BCG vaccination increased the amount of fibrosis in low grade granulomas. BCG vaccination did not influence the amount of mineralization. Mineralization increased with the severity of the lesion.

Fibrosis was the only metric in which experimental group had a significant effect. The primary difference between the two groups of animals used for this study is age, where the older cohort (from Experiment 1) had higher average fibrosis than the younger animals (from Experiment 2) (data not shown). That being said, there were no significant differences in the percentage of fibrosis per granuloma between vaccinates and non-vaccinates for either experimental group (Figure 6A). Collectively, when evaluated by grade, low grade granulomas of vaccinates had a significantly (p = 0.0344) higher percentage of fibrosis than low-grade granulomas of non-vaccinates (Figure 6B). When mineralization was evaluated, no significant differences were observed in the percentage of mineralization between vaccinates and non-vaccinates (Figure 7A). Additionally, mineralization was similar within grades of granuloma, despite different vaccination groups (Figure 7B). Significant differences existed between low grade granulomas and their corresponding high-grade granulomas with a p=0.0009 in vaccinates and p < 0.0001 in non-vaccinates.”

The corrected sentences appear below:

“-BCG vaccination did not alter the levels of fibrosis or mineralization within granulomas. Mineralization generally increased with the severity of the lesion.

There were no significant differences in the percentage of fibrosis per granuloma between vaccinates and non-vaccinates for either experimental group (Figure 6A). This was also true when fibrosis was analyzed by grade (Figure 6B). When mineralization was evaluated, no significant differences were observed in the percentage of mineralization in granulomas from vaccinates and non-vaccinates (Figure 7A) and mineralization was similar between vaccinates and non-vaccinates when evaluating the same grade of granuloma (Figure 7B). In E1, granulomas from vaccinates and non-vaccinates had significantly more mineralization in the high-grade granulomas compared to the low-grade granulomas (*p* = 0.0396 for vaccinates and *p* = 0.0076 for non-vaccinates). In contrast, in E2 only granulomas from non-vaccinates had high-grade granulomas with significantly higher mineralization than low-grade lesions (*p* = 0.0006).”

A correction has been made to **Discussion**, 1. This section previously stated:

“This study utilized 88 pulmonary lymph nodes from 44 animals (22 BCG vaccinates, 22 non-vaccinates), resulting in the evaluation of a total of 1,770 granulomas. Of these, 417 granulomas were in BCG vaccinates while 1353 were in non-vaccinates.”

The corrected sentences appear below:

“This study examined 418 granulomas from BCG-vaccinates (144 from E1 and 274 from E2) and 1,353 from non-vaccinates (702 from E1 and 651 from E2). We looked at the size of the granuloma, amount of necrosis, number of multinucleated giant cells, the amount of fibrosis and the amount of mineralization.”

A correction has been made to **Discussion**, 3. This section previously stated:

“As expected, and in accordance with previous studies, BCG decreased the lesion burden (Buddle et al., 1995; Johnson et al., 2006; Canto Alarcon et al., 2013; Dean et al., 2014; Salguero et al., 2017; Palmer et al., 2022). Vaccinated animals had an average of 19 granulomas per tissue section while non-vaccinated animals averaged 62 granulomas. Interestingly, despite this contrast, the relative breakdown of high- and low-grade granulomas did not significantly vary, with vaccinates and non-vaccinates having similar percentages of each. This suggests that the protective nature of BCG is, in part, by preventing the establishment of granulomas as opposed to necessarily preventing their progression to a higher grade. These findings are consistent with Johnson et. al and studies performed in white-tailed deer which report all stages of granulomas in lymph nodes of BCG vaccinates (Johnson et al., 2006; Nol et al., 2008; Palmer et al., 2009). Although BCG did not prevent high grade granulomas from being formed, it maintained smaller lesions on average with less necrosis.”

The corrected sentences appear below:

“BCG decreased the lesion burden in both groups, which is in accordance to previous studies (Buddle et al., 1995; Johnson et al., 2006; Canto Alarcon et al., 2013; Dean et al., 2014; Salguero et al., 2017; Palmer et al., 2022). When evaluated as a whole, E1 vaccinated animals had an average of 11.9 granulomas per tissue section while non-vaccinated animals averaged 63.8. In E2, vaccinated animals averaged 27.4 granulomas per tissue section while non-vaccinates averaged 59.2. Interestingly, the relative breakdown of high- and low-grade granulomas did not significantly vary, with vaccinates and non-vaccinates having similar percentages of each. This suggests that the protective nature of BCG is, in part, by preventing the establishment of granulomas as opposed to necessarily preventing their progression to a higher grade. These findings are consistent with Johnson et al. and studies performed in white-tailed deer which report all stages of granulomas in lymph nodes of BCG vaccinates (Johnson et al., 2006; Nol et al., 2008; Palmer et al., 2009).

Although BCG did not prevent high grade granulomas from being formed, in E2 animals it maintained smaller lesions on average and, more specifically, within the high-grade granulomas. In E1 there were no significant differences in average granuloma area, reflecting an aspect of the variability seen within BCG vaccinates. In both E1 and E2, high-grade granulomas averaged larger areas than low-grade granulomas, irrespective of vaccination status. In both E1 and E2, vaccinates had less necrosis within lesions than non-vaccinates. In E1high-grade granulomas of non-vaccinates had more necrosis than vaccinates.”

A correction has been made to **Discussion**, 4. This section previously stated:

“Fibrosis was the single metric in which there were statistical differences between experimental cohorts, of which the primary difference between the groups is age; older animals of Experiment 1 and the younger animals of Experiment 2. Older animals from Experiment 1 averaged more fibrosis in granulomas compared to younger animals of Experiment 2 (data not shown). This was unexpected and warrants further investigation, as it has not been reported previously. A possible explanation for the difference in fibrosis found between the two age groups could be levels of tumor growth factor beta (TGF-β), the cytokine most responsible for stimulating fibrosis. However, according to a study performed in sheep, TGF-β is lower in older animals vs neonates(Sow et al., 2012). This would suggest other cytokines and/or immunological mechanisms are contributing to these differences. It has been demonstrated that calves vaccinated at birth with BCG produce similar levels of interferon gamma (IFN-γ) as calves 5-8 months old, however unlike the older calves, the neonatal vaccinates had a gradual decline in IFN-γ levels(Buddle et al., 2003). This might be relevant as IFN-γ can suppress TGF-β secretion by macrophages and could ultimately be responsible for the differences in fibrosis seen between age groups(Warsinske et al., 2017b). It is also possible that the older animals, being exposed for a longer period to the outdoors, may have come into contact with environmental mycobacteria, which altered their fibrotic response when exposed to virulent M. bovis (Buddle et al., 2003). Regardless, when taken as a collective group, vaccinates and non-vaccinates had similar overall percentages of fibrosis, a finding in contrast to previous studies (Johnson et al., 2006; Salguero et al., 2017).

And, “When broken down by vaccinate group, the amount of fibrosis was significantly higher in low grade granulomas of vaccinates than non-vaccinates. By the time lesions progressed to high-grade, this significance was lost.”

These statements have been removed.

A correction has been made to **Discussion**, 4. This section previously stated:

“However, in light of those studies, the finding of increased fibrosis in the low-grade lesions of vaccinates is intriguing and may represent a rapid immunological response as a result of BCG-induced memory, and an attempt at early containment of the infection.”

The corrected sentences appear below:

“Surprisingly, there were no significant differences in the percentage of fibrosis within granulomas between vaccinates or non-vaccinates, regardless of granuloma grade. This is a finding in contrast to other studies (Johnson et al., 2006; Salguero et al., 2017).”

And, “Our findings show BCG does not impart protection by increasing fibrosis. Further studies evaluating the amount of central fibrosis compared to peripheral fibrosis may identify effects of BCG.”

A correction has been made to **Discussion**, 5. This section previously stated:

“Dystrophic mineralization is a common sequela to necrosis and therefore it is not surprising to see levels of mineralization increased in high grade granulomas. Nevertheless, it is interesting to note that the percentage of mineralization between high-grade granulomas were similar between vaccination groups and that small amounts of mineralization were present in the low-grade granulomas.”

The corrected sentences appear below:

Dystrophic mineralization is a common sequela to necrosis and therefore it is not surprising to see levels of mineralization increased in high grade granulomas of both E1 and E2, independent of vaccination status. Nevertheless, it is interesting to note that the overall percentage of mineralization between vaccinates and non-vaccinates was non-significant. Also of note is that small amounts of mineralization were present in the low-grade granulomas.

A correction has been made to **Discussion**, 6. This section previously stated:

“In this study we found no significant differences between groups or grade, however there was a trend for non-vaccinates to have more MNGC in their high-grade granulomas compared to their low-grade granulomas, suggesting increased bacterial burden may have played a role.”

The corrected sentence appears below:

“Unfortunately, in both E1 and E2 we found no significant differences between granuloma grades regardless of vaccination.”

A correction has been made to **Discussion**, 8. This section previously stated:

“Using banked samples from two previous experiments meant that treatments between the two experiments were not identical. The vaccination dose and challenge dose varied slightly between experimental groups, however as these were < 1 log, we felt it was an acceptable range. Enumeration of Mycobacterium bovis in liquid culture is difficult due to the propensity the bacilli to clump (Gautam et al., 2022).”

The corrected sentences appear below:

“Although not the objective of this study, the use of banked samples from two separate experiments has highlighted an important aspect of BCG and *M. bovis* research. While we expected both experimental groups to behave similarly, in fact there were 3 parameters where only one experimental group had significance between BCG vaccinates and non-vaccinates (granuloma and MNGC numbers in E1 and average area in E2). Experimental groups varied based on age of vaccination, dose of BCG and strain and dose of challenge. It would be helpful to directly compare each of these variables to determine how each affects resulting lesions.”

And, “Nevertheless, these authors suggest vaccinating and challenging animals the same day or utilizing the same seed stocks to eliminate these variabilities.”

A correction has been made to **Discussion**, 10. This section previously stated:

“Despite this fact, BCG vaccinates have smaller granulomas with less tissue destruction than non-vaccinates. Fibrosis was higher in low-grade granulomas of vaccinates compared to non-vaccinates but this significance was lost when grade was excluded. Mineralization and MNGC density were similar between vaccinates and non-vaccinates, suggesting these may be vaccine-independent events.”

The corrected sentences appear below:

“BCG may reduce tissue destruction as not only did E2 vaccinate granulomas have a smaller average size, but necrosis was reduced in granulomas of both E1 and E2 vaccinates. The role of BCG vaccination in altering MNGC density is unclear as numbers were reduced in E1, but significance was lost when evaluating by grade. Finally, fibrosis and mineralization were not altered by BCG vaccination in either experimental group suggesting these may be vaccine-independent events.”

The authors apologize for these errors. The original article has been updated.

